# 3,3′-Dibromo-1,1′-[(propane-1,3-diyl­dioxy)­bis(nitrilo­methyl­idyne)]dibenzene

**DOI:** 10.1107/S1600536808018187

**Published:** 2008-06-21

**Authors:** Wen-Kui Dong, Yu-Jie Ding, Ya-Ling Luo, Zhong-Wu Lv, Li Wang

**Affiliations:** a School of Chemical and Biological Engineering, Lanzhou Jiaotong University, Lanzhou 730070, People’s Republic of China; b Department of Biochemical Engineering, Anhui University of Technology And Science, Wuhu 241000, People’s Republic of China

## Abstract

The mol­ecule of the title compound, C_17_H_16_Br_2_N_2_O_2_, lies on a twofold axis that passes through the middle atom of the three-atom trimethyl­ene unit. The two aromatic rings are aligned at an angle of 76.02 (4)°.

## Related literature

For similar Schiff bases, see: Aysegul *et al.* (2005 ▶); Cordes & Jencks (1962 ▶); Dong *et al.* (2008 ▶); Duan *et al.* (2007 ▶); Shi *et al.* (2007 ▶); Koehler *et al.* (1964 ▶).
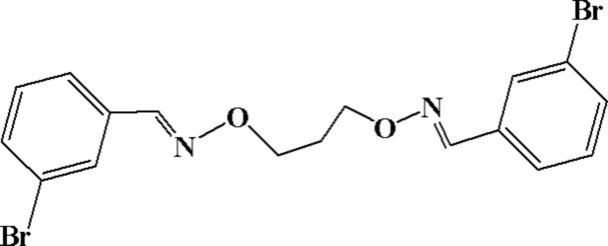


## Experimental

### 

#### Crystal data

C_17_H_16_Br_2_N_2_O_2_
*M* *_r_* = 440.14
Monoclinic, 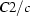

*a* = 24.397 (3) Å
*b* = 4.4848 (4) Å
*c* = 17.189 (2) Å
β = 114.009 (2)°
*V* = 1718.0 (3) Å^3^
*Z* = 4
Mo *K*α radiation
μ = 4.73 mm^−1^
*T* = 298 (2) K
0.48 × 0.35 × 0.24 mm

#### Data collection

Bruker SMART 1000 CCD area-detector diffractometer
Absorption correction: multi-scan (*SADABS*; Sheldrick, 1996 ▶) *T*_min_ = 0.210, *T*_max_ = 0.397 (expected range = 0.170–0.321)
3683 measured reflections
1497 independent reflections
1179 reflections with *I* > 2σ(*I*)
*R*_int_ = 0.094

#### Refinement

*R*[*F* ^2^ > 2σ(*F* ^2^)] = 0.050
*wR*(*F* ^2^) = 0.133
*S* = 1.07
1497 reflections
105 parameters
H-atom parameters constrained
Δρ_max_ = 0.60 e Å^−3^
Δρ_min_ = −0.66 e Å^−3^

### 

Data collection: *SMART* (Bruker, 1996 ▶); cell refinement: *SAINT* (Bruker, 1996 ▶); data reduction: *SAINT*; program(s) used to solve structure: *SHELXS97* (Sheldrick, 2008 ▶); program(s) used to refine structure: *SHELXL97* (Sheldrick, 2008 ▶); molecular graphics: *SHELXTL* (Sheldrick, 2008 ▶); software used to prepare material for publication: *SHELXTL*.

## Supplementary Material

Crystal structure: contains datablocks global, I. DOI: 10.1107/S1600536808018187/ng2462sup1.cif

Structure factors: contains datablocks I. DOI: 10.1107/S1600536808018187/ng2462Isup2.hkl

Additional supplementary materials:  crystallographic information; 3D view; checkCIF report

## Figures and Tables

**Fig. 1.**
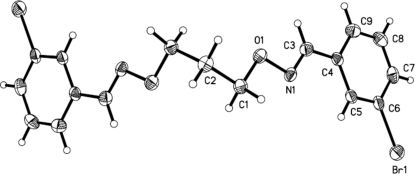
The molecule structure of (I) with atom numbering. Displacement ellipsoids for non-hydrogen atoms are drawn at the 30% probability level.

## supplementary crystallographic information

### Comment

Schiff base compounds have been widely used as versatile ligands involved in various metal chelations to form transition metal complexes with interesting properties (Aysegul *et al.*, 2005; Dong *et al.*, 2008). Although most of Schiff base derivatives are stable in solution and in solid state, C=N bonds often suffer exchange reaction (Koehler *et al.*, 1964) as well as hydrolysis (Cordes & Jencks, 1962). Rate constants of oxime formation are smaller than those of imine formation and the equilibrium constants are larger by several orders. Hence, bisoxime-type compound should be stable enough to resist the metathesis of the C=N bonds. In this paper, a novel ligand, 3,3'-dibromo-1,1'-[propane-1,3-diyldioxybis(nitrilomethylidyne)]dibenzene (I) was designed and synthesized, and shown in Fig. 1.

The single-crystal structure of (I) is built up by discrete C_17_H_16_Br_2_N_2_O_2_ molecules, in which all bond lengths are in normal ranges. There is a crystallographic twofold rotation axis passing through the middle point (symmetry code: -*x*, *y*, 1/2 - *z*) of the C—C unit. The molecule adopts a *trans* conguration in which two benzane rings are apart from each other and form a dihedral angle of 76.02 (4) Å. The oxime, bromo groups of (I) lie in *trans* positions relative to the middle point in the N—O—CH_2_—CH_2_—O—N linkage, which is similar to what is observed in our previously reported salen-type bisoxime compound of 2,2'-[(propane-1,3-diyldioxy)bis(nitrilomethylidyne)]diphenol (Duan *et al.*, 2007). The molecule exhibits a zigzag chain array along *a* axis.

### Experimental

3,3'-Dibromo-1,1'-[propane-1,3-diyldioxybis(nitrilomethylidyne)]dibenzene (I) was synthesized according to an analogous method reported earlier (Shi *et al.*, 2007). To an ethanol solution (2 ml) of 3-bromo-benzaldehyde (283.0 mg, 1.48 mmol) was added an ethanol solution (3 ml) of 1,3-bis(aminooxy)propane (78.6 mg, 0.74 mmol). The mixed solution was stirred at 328 K for 6 h. The precipitate was filtered, and washed successively with ethanol and ethanol-hexane (1:4), respectively. The product was dried under vacuum to yield 157.5 mg of (I). Yield, 48.3%. mp. 350.5–352.5 K. Anal. Calc. for C_17_H_16_Br_2_N_2_O_2_: C, 45.76; H, 3.49; N, 6.47. Found: C, 45.66; H, 3.43; N, 6.29.

Colorless needle-like single crystals suitable for X-ray diffraction studies were obtained after several weeks by slow evaporation from a methanol-tetrahydrofuran-ethyl acetate mixed solution of (I).

### Refinement

Non-H atoms were refined anisotropically. H atoms were treated as riding atoms with distances C—H = 0.97 (CH_2_), 0.93 Å (CH), and *U*_iso_(H) = 1.2 *U*_eq_(C) and 1.5 *U*_eq_(O).

### Crystal data

**Table:**

C_17_H_16_Br_2_N_2_O_2_	*F*_000_ = 872
*M**_r_* = 440.14	*D*_x_ = 1.702 Mg m^−^^3^
Monoclinic, *C*2/*c*	Mo *K*α radiation λ = 0.71073 Å
Hall symbol: -C 2yc	Cell parameters from 2255 reflections
*a* = 24.397 (3) Å	θ = 2.5–27.9º
*b* = 4.4848 (4) Å	µ = 4.73 mm^−^^1^
*c* = 17.189 (2) Å	*T* = 298 (2) K
β = 114.009 (2)º	Rod, colorless
*V* = 1718.0 (3) Å^3^	0.48 × 0.35 × 0.24 mm
*Z* = 4	

### Data collection

**Table:**

Bruker SMART 1000 CCD area-detector diffractometer	1497 independent reflections
Radiation source: fine-focus sealed tube	1179 reflections with *I* > 2σ(*I*)
Monochromator: graphite	*R*_int_ = 0.094
*T* = 298(2) K	θ_max_ = 25.0º
φ and ω scans	θ_min_ = 1.8º
Absorption correction: multi-scan(SADABS; Sheldrick, 1996)	*h* = −27→28
*T*_min_ = 0.210, *T*_max_ = 0.397	*k* = −5→5
3683 measured reflections	*l* = −20→15

### Refinement

**Table:**

Refinement on *F*^2^	Secondary atom site location: difference Fourier map
Least-squares matrix: full	Hydrogen site location: inferred from neighbouring sites
*R*[*F*^2^ > 2σ(*F*^2^)] = 0.050	H-atom parameters constrained
*wR*(*F*^2^) = 0.133	*w* = 1/[σ^2^(*F*_o_^2^) + (0.061*P*)^2^] where *P* = (*F*_o_^2^ + 2*F*_c_^2^)/3
*S* = 1.07	(Δ/σ)_max_ = 0.001
1497 reflections	Δρ_max_ = 0.60 e Å^−^^3^
105 parameters	Δρ_min_ = −0.66 e Å^−^^3^
Primary atom site location: structure-invariant direct methods	Extinction correction: none

### Special details

**Table:**

Geometry. All e.s.d.'s (except the e.s.d. in the dihedral angle between two l.s. planes) are estimated using the full covariance matrix. The cell e.s.d.'s are taken into account individually in the estimation of e.s.d.'s in distances, angles and torsion angles; correlations between e.s.d.'s in cell parameters are only used when they are defined by crystal symmetry. An approximate (isotropic) treatment of cell e.s.d.'s is used for estimating e.s.d.'s involving l.s. planes.
Refinement. Refinement of *F*^2^ against ALL reflections. The weighted *R*-factor *wR* and goodness of fit *S* are based on *F*^2^, conventional *R*-factors *R* are based on *F*, with *F* set to zero for negative *F*^2^. The threshold expression of *F*^2^ > σ(*F*^2^) is used only for calculating *R*-factors(gt) *etc*. and is not relevant to the choice of reflections for refinement. *R*-factors based on *F*^2^ are statistically about twice as large as those based on *F*, and *R*- factors based on ALL data will be even larger.

### Fractional atomic coordinates and isotropic or equivalent isotropic displacement parameters (Å^2^)

**Table:**

	*x*	*y*	*z*	*U*_iso_*/*U*_eq_	Occ. (<1)
Br1	0.78652 (2)	1.12437 (15)	0.11896 (3)	0.0608 (3)	
O1	0.53639 (12)	0.3149 (7)	−0.13426 (18)	0.0393 (8)	
N1	0.58506 (15)	0.4899 (10)	−0.0798 (2)	0.0352 (9)	
C1	0.5538 (2)	0.1605 (10)	−0.1934 (3)	0.0369 (11)	
H1A	0.5876	0.0299	−0.1635	0.044*	
H1B	0.5653	0.3011	−0.2270	0.044*	
C2	0.5000	−0.0186 (16)	−0.2500	0.0371 (15)	
H2A	0.4880	−0.1465	−0.2142	0.044*	0.50
H2B	0.5120	−0.1465	−0.2858	0.044*	0.50
C3	0.5704 (2)	0.6276 (10)	−0.0265 (3)	0.0398 (12)	
H3	0.5317	0.6039	−0.0296	0.048*	
C4	0.6117 (2)	0.8206 (10)	0.0390 (3)	0.0350 (11)	
C5	0.6701 (2)	0.8759 (10)	0.0463 (3)	0.0368 (11)	
H5	0.6836	0.7885	0.0083	0.044*	
C6	0.7070 (2)	1.0573 (11)	0.1091 (3)	0.0391 (12)	
C7	0.6887 (2)	1.1924 (12)	0.1666 (3)	0.0472 (13)	
H7	0.7144	1.3178	0.2087	0.057*	
C8	0.6313 (3)	1.1373 (11)	0.1600 (3)	0.0504 (14)	
H8	0.6185	1.2245	0.1988	0.061*	
C9	0.5930 (2)	0.9556 (12)	0.0972 (3)	0.0444 (13)	
H9	0.5544	0.9222	0.0935	0.053*	

### Atomic displacement parameters (Å^2^)

**Table:**

	*U*^11^	*U*^22^	*U*^33^	*U*^12^	*U*^13^	*U*^23^
Br1	0.0451 (4)	0.0844 (6)	0.0468 (4)	−0.0215 (3)	0.0126 (3)	−0.0037 (3)
O1	0.0346 (18)	0.042 (2)	0.0377 (19)	−0.0112 (15)	0.0110 (15)	−0.0071 (16)
N1	0.030 (2)	0.036 (2)	0.033 (2)	−0.0059 (17)	0.0067 (17)	0.0016 (19)
C1	0.038 (3)	0.036 (3)	0.035 (2)	0.003 (2)	0.014 (2)	0.007 (2)
C2	0.039 (4)	0.026 (4)	0.043 (4)	0.000	0.013 (3)	0.000
C3	0.037 (3)	0.040 (3)	0.039 (3)	−0.009 (2)	0.012 (2)	0.002 (2)
C4	0.040 (3)	0.035 (3)	0.029 (2)	0.000 (2)	0.013 (2)	0.009 (2)
C5	0.040 (3)	0.041 (3)	0.027 (2)	−0.001 (2)	0.012 (2)	0.003 (2)
C6	0.043 (3)	0.044 (3)	0.026 (2)	−0.006 (2)	0.009 (2)	0.005 (2)
C7	0.059 (3)	0.042 (3)	0.030 (2)	−0.007 (3)	0.008 (2)	−0.005 (2)
C8	0.066 (4)	0.052 (4)	0.041 (3)	0.001 (3)	0.029 (3)	−0.007 (3)
C9	0.047 (3)	0.040 (3)	0.050 (3)	0.000 (2)	0.024 (3)	0.001 (3)

### Geometric parameters (Å, °)

**Table:**

Br1—C6	1.900 (4)	C3—H3	0.9300
O1—N1	1.413 (4)	C4—C9	1.396 (6)
O1—C1	1.431 (5)	C4—C5	1.400 (6)
N1—C3	1.270 (6)	C5—C6	1.359 (6)
C1—C2	1.508 (6)	C5—H5	0.9300
C1—H1A	0.9700	C6—C7	1.381 (6)
C1—H1B	0.9700	C7—C8	1.379 (7)
C2—C1^i^	1.508 (6)	C7—H7	0.9300
C2—H2A	0.9700	C8—C9	1.372 (7)
C2—H2B	0.9700	C8—H8	0.9300
C3—C4	1.453 (6)	C9—H9	0.9300
			
N1—O1—C1	109.1 (3)	C9—C4—C3	119.2 (4)
C3—N1—O1	109.9 (3)	C5—C4—C3	122.3 (4)
O1—C1—C2	106.5 (3)	C6—C5—C4	120.0 (4)
O1—C1—H1A	110.4	C6—C5—H5	120.0
C2—C1—H1A	110.4	C4—C5—H5	120.0
O1—C1—H1B	110.4	C5—C6—C7	121.8 (4)
C2—C1—H1B	110.4	C5—C6—Br1	119.3 (3)
H1A—C1—H1B	108.6	C7—C6—Br1	118.9 (4)
C1—C2—C1^i^	115.7 (5)	C8—C7—C6	118.5 (5)
C1—C2—H2A	108.4	C8—C7—H7	120.8
C1^i^—C2—H2A	108.4	C6—C7—H7	120.8
C1—C2—H2B	108.4	C9—C8—C7	121.0 (4)
C1^i^—C2—H2B	108.4	C9—C8—H8	119.5
H2A—C2—H2B	107.4	C7—C8—H8	119.5
N1—C3—C4	122.6 (4)	C8—C9—C4	120.3 (4)
N1—C3—H3	118.7	C8—C9—H9	119.8
C4—C3—H3	118.7	C4—C9—H9	119.8
C9—C4—C5	118.4 (4)		
			
C1—O1—N1—C3	−179.7 (4)	C4—C5—C6—C7	−0.3 (7)
N1—O1—C1—C2	−179.4 (4)	C4—C5—C6—Br1	179.0 (3)
O1—C1—C2—C1^i^	65.8 (3)	C5—C6—C7—C8	0.7 (7)
O1—N1—C3—C4	178.7 (4)	Br1—C6—C7—C8	−178.6 (4)
N1—C3—C4—C9	−177.6 (4)	C6—C7—C8—C9	−0.8 (8)
N1—C3—C4—C5	2.1 (7)	C7—C8—C9—C4	0.6 (8)
C9—C4—C5—C6	0.0 (6)	C5—C4—C9—C8	−0.1 (7)
C3—C4—C5—C6	−179.7 (4)	C3—C4—C9—C8	179.6 (5)

Symmetry codes: (i) −*x*+1, *y*, −*z*−1/2.

### Hydrogen-bond geometry (Å, °)

**Table:**

*D*—H···*A*	*D*—H	H···*A*	*D*···*A*	*D*—H···*A*
C1—H3···C3#	0.93	2.36	3.189	148
